# Current advances in experimental and computational approaches to enhance CAR T cell manufacturing protocols and improve clinical efficacy

**DOI:** 10.3389/fmmed.2024.1310002

**Published:** 2024-02-01

**Authors:** Alfredo S. Colina, Viren Shah, Ravi K. Shah, Tanya Kozlik, Ranjan K. Dash, Scott Terhune, Anthony E. Zamora

**Affiliations:** ^1^ Department of Microbiology & Immunology, Medical College of Wisconsin, Milwaukee, WI, United States; ^2^ Department of Biomedical Engineering, Medical College of Wisconsin and Marquette University, Milwaukee, WI, United States; ^3^ Department of Medicine, Medical College of Wisconsin, Milwaukee, WI, United States

**Keywords:** chimeric antigen receptor T cells, CAR T cell, manufacturing, functional assays, computational modeling

## Abstract

Since the FDA’s approval of chimeric antigen receptor (CAR) T cells in 2017, significant improvements have been made in the design of chimeric antigen receptor constructs and in the manufacturing of CAR T cell therapies resulting in increased *in vivo* CAR T cell persistence and improved clinical outcome in certain hematological malignancies. Despite the remarkable clinical response seen in some patients, challenges remain in achieving durable long-term tumor-free survival, reducing therapy associated malignancies and toxicities, and expanding on the types of cancers that can be treated with this therapeutic modality. Careful analysis of the biological factors demarcating efficacious from suboptimal CAR T cell responses will be of paramount importance to address these shortcomings. With the ever-expanding toolbox of experimental approaches, single-cell technologies, and computational resources, there is renowned interest in discovering new ways to streamline the development and validation of new CAR T cell products. Better and more accurate prognostic and predictive models can be developed to help guide and inform clinical decision making by incorporating these approaches into translational and clinical workflows. In this review, we provide a brief overview of recent advancements in CAR T cell manufacturing and describe the strategies used to selectively expand specific phenotypic subsets. Additionally, we review experimental approaches to assess CAR T cell functionality and summarize current *in silico* methods which have the potential to improve CAR T cell manufacturing and predict clinical outcomes.

## Introduction

Over the last few decades, efforts exploiting the immune system to target and eliminate malignant cells have grown in popularity and clinical utility. Recently, immunotherapeutic approaches have focused on redirecting T cells to preferentially target specific antigens expressed on cancerous cells. One of the most widely adopted approaches consists of using T cells engineered to express surface-bound synthetic chimeric antigen receptors (CARs). Since the first commercial authorization by the United States Food and Drug Administration (FDA) and European Medicine Agency (EMA) in 2017 and 2018, respectively, advancements in the genetic modification of CAR T cells have continued to progress at an astonishing rate (European Medicines Agency, 2018b; European Medicines Agency, 2018c; FDA, 2021a; FDA, 2021b). Each new generation of CAR T cell has focused on incorporating increasingly sophisticated engineering strategies into the CAR construct with the hope of driving greater persistence of CAR T cells in patients and better clinical efficacy. Yet, despite the constant evolution in CAR T cell design, a sizeable fraction of cancer patients will ultimately relapse after treatment and succumb to their disease and major barriers exist before comparable successes will be realized in the solid tumor setting (Park et al., 2018; Byrne et al., 2019; Locke et al., 2019; Xu et al., 2019; Melenhorst et al., 2022). As a result, recent efforts in CAR T manufacturing have focused on addressing lingering obstacles related to enhancing CAR T cell persistence, decreasing CAR-mediated toxicity, and maintaining cells in a highly functional state post-infusion.

**FIGURE 1:**
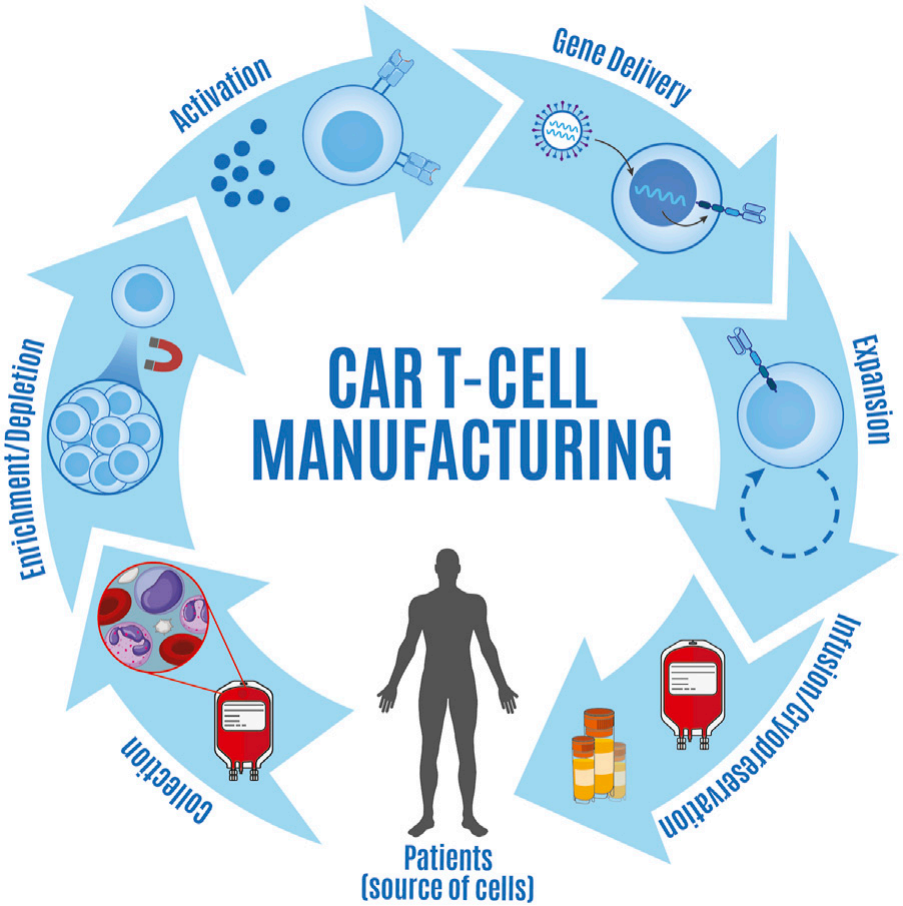
Overview of steps involved in CAR T cell manufacturing. CAR T cell manufacturing consists of a series of steps including collection of apheresis products from patients (source of cells), enrichment and/or depletion of specific cell types, T cell activation, gene delivery, CAR T cell expansion, and either cryopreserva-tion prior to infusion or infusion of fresh product.

While opportunities to continue improving CAR T cell performance by means of modifying or introducing new domains to the CAR construct remain, a recent switch in focus towards revisiting how we manufacture CAR T cells to address ongoing challenges is beginning to take center stage. Growing efforts aim to target one or more of the key steps along the CAR T cell manufacturing continuum (Figure 1) in hopes of generating a superior product with favorable biological attributes. Fueled by retrospective studies from the clinical application of CAR T cells, it is becoming clear that differences in the manufacturing process can directly impact the composition of the resulting product including the percent of cells expressing CARs, CD4:CD8 T cell ratios, and the relative frequency of T cells at specific stages of differentiation (Zurko et al., 2021). Importantly, the differentiation status of CAR T cells has been shown to impact post-infusion expansion and persistence due to inherent biological differences between the subsets (Wherry et al., 2003; Louis et al., 2011; Schmueck-Henneresse et al., 2017; Fraietta et al., 2018; Philip and Schietinger, 2019). Several aspects of the manufacturing process can be coopted to direct the cellular products toward optimal composition for improving therapeutic response (Gargett et al., 2019; Stock et al., 2019). These include methods of introducing genetic constructs into T lymphocytes, the selection of culture media additives to support cell growth, the activation and expansion of CAR T cells (including new activation agents and considering the duration of expansion), and the selection or depletion of specific subgroups of cells (Stemberger et al., 2012; Riddell et al., 2014; Schmueck-Henneresse et al., 2017; Xu et al., 2018; K et al., 2022). While we acknowledge that the discovery of novel CAR constructs and antigenic targets play an important role in the continuous success of this therapy (Holzinger and Abken, 2019), detailed review of these topics are outside the scope of this review and have been covered elsewhere (Sadelain et al., 2009; van der Stegen et al., 2015; Holzinger and Abken, 2019; Abken, 2021). The goal of this review is to cover specific steps of the CAR T cell manufacturing process and describe how changes in these steps can help address some of the ongoing challenges with CAR T cell efficacy. Furthermore, given that each step is manipulatable, we anticipate that by altering, optimizing, testing, and modeling these steps using *in vitro* assays and computational models, we will glean important biological insight that will ultimately impact clinical outcome.

To gain insight into how CAR T cells function once administered to patients with cancer, we must continue to develop better methods to assess product functionality (cytolytic ability, proliferation capacity, and cytokine production) and determine whether the phenotypic and functional trajectories seen *in vitro* recapitulate what occurs *in vivo*. By linking how specific perturbations of cellular activation alter the phenotypic and functional signatures of CAR T cells, we may better establish how these correlate with therapeutic efficacy. In this review, we will overview specific assays that can be used to measure cytokine secretion and cytotoxicity and how they play a vital role in determining pre-infusion CAR T cell function (FDA, 2011; FDA, 2023b). We also introduce how computational methods are being used as tools to help predict CAR T cell function and clinical efficacy by incorporating variables such as CAR surface expression, binding affinity between CAR and antigen, and product differentiation status. Overall, through identifying the relevant manufacturing variables and then detailing current experimental and computational methods that can inform the optimization of these variables, our aim is to contribute to the discourse on how to best modulate CAR T cell manufacturing to improve therapeutic responses.

## Manufacturing approaches for CAR T-cells

The major steps for all current CAR T cell manufacturing processes include: i) collection of apheresis product, ii) enrichment and/or depletion of specific cell types, iii) T cell activation, iv) gene delivery, v) CAR T cell expansion, vi) CAR T cell formulation and filing, and vii) either cryopreservation prior to infusion or infusion of fresh product. Many of the guiding principles for this process have been shaped by the development of tumor-infiltrating lymphocytes (TIL) as therapeutic agents, which have historically implemented a similar isolation, culturing, and expansion strategy (Rosenberg et al., 1990). As early considerations for infusion primarily relied on ensuring sufficient numbers of CAR-expressing cells were obtained for infusion, the duration and formulations used during the manufacturing process often varied and resulted in inconsistent clinical responses across clinical trials (Wang and Rivière, 2016; Gajra et al., 2022). Given the lack of activation and expansion of first-generation CAR T cells, initial emphasis was placed on developing newer generations of constructs that would circumvent these shortcomings (Boyiadzis et al., 2018; Guedan et al., 2018; Sermer and Brentjens, 2019). While second and third generation CAR constructs have displayed increased cell expansion (Li et al., 2017; Weinkove et al., 2019), the clinical results have varied greatly between design iterations. Recent studies point to the variegated cellular differentiation status amongst products leading to heterogenous responses between individuals; however, more universal is the data supporting less differentiated cells having increased function and persistence (Fraietta et al., 2018; Hoffmann et al., 2018). As a result, optimizing the manufacturing process in CAR T cell product generation is now a significant goal for translational researchers and clinicians alike and product manufacturing has been identified as a key factor that drives functional differences between infused products (Gargett et al., 2019; Stock et al., 2019; Tyagarajan et al., 2020).

### Media composition

The composition of the culturing media plays a critical role in determining the differentiation and functional status of transduced cells during the activation and expansion steps of CAR T cell manufacturing. Recent findings have emphasized the utility of selecting culture conditions that minimize the length of manufacturing and at the same time yield high absolute numbers of functionally relevant subpopulations responsible for mediating tumor control (Ghassemi et al., 2018; Ghassemi et al., 2022). Specifically, previous analyses looking at the composition of infused cells have determined that cells expressing surface proteins such as CD27^+^, CCR7+, and CD45RA + are less differentiated and belong to subgroups of T cells, such as central memory (T_CM_) and naive (T_N_) subsets, with greater proliferative capacity and persistent antitumor function (Gattinoni et al., 2012; Busch et al., 2016; Stock et al., 2019).

Recent comparative studies using commercially available T cell growth media formulations, such as RPMI-1640, Optimizer™, X-VIVO 15™, and TexMACS™, have examined how the choice of base media can influence the expansion of CAR T cells. The findings suggest that media selection should be based on which T cell subsets are desired at the end of manufacturing as different media lead to varied phenotypes and functions of the T cells (Sato et al., 2009; Lu et al., 2016). In addition to choosing an appropriate base medium, media additives can also alter the end product. Specific sources of serum significantly impact the potency and proliferation of CAR T cells (Gardner et al., 2017; Fraietta et al., 2018; Ghassemi et al., 2020), and consideration of xeno- and serum-free media can lead to increased viability and reduced T cell exhaustion (Sanyanusin et al., 2023; Sartorius, 2023). Addition of specific supportive cytokines during cell activation and expansion can also drastically alter the relative frequency of T cells across the spectrum of differentiation states (Gargett and Brown, 2015; Gong et al., 2019; Zhang X. et al., 2022). While many commercial products rely on interleukin (IL)-2 as the sole supportive growth factor, emerging studies have highlighted the benefits of replacing IL-2 with IL-7 and IL-15 and have shown that the later cytokines result in a final cell product with a less differentiated phenotype and enhanced therapeutic potential (Cieri et al., 2013; Alizadeh et al., 2019; Zhou et al., 2019; Battram et al., 2021; Kim et al., 2022).

To reduce the frequency of terminally differentiated cells, others have sought to inhibit specific metabolic processes via the alteration of cell signaling processes (Zheng et al., 2018; Li W. et al., 2023). The addition of Protein kinase B (AKT) inhibitors during the manufacturing of CD19-specific CAR T cells has been shown to increase antitumor function by blocking phosphatidylinositol 3-kinase (PI3K) signaling while maintaining mitogen-activated protein kinase (MAPK) signaling, leading to accumulation of T_CM_ associated transcription factors such as FOXO1 (Klebanoff et al., 2017; Urak et al., 2017; Coleman et al., 2021; Delpoux et al., 2021). Another strategy for improving the quantity and quality of memory T cells is to target the mammalian target of rapamycin (mTOR) and IL-2 receptor signaling pathways. Previous studies have shown that introduction of rapamycin during manufacturing of T cell products can enhance T cell functionality, increase T cell viability and resistance to apoptosis, and alter the metabolic state of activated T cells by partially inhibiting mTOR (Araki et al., 2010; Schmueck et al., 2012; Alizadeh et al., 2019; Amini et al., 2019). Taking a different approach, the addition of Wnt/β-catenin signaling pathway modulators has been shown to impede the transition of naïve to effector T cell subsets, which may provide additional avenues to increase the frequency of less differentiated and highly functional CAR T cell products (Gattinoni et al., 2009; Muralidharan et al., 2011; Kondo et al., 2018). Overall, these studies highlight the importance of carefully considering and optimizing the composition of culturing media to expand functionally potent CAR T cells.

### Isolation and depletion of select subsets

The isolation and/or depletion of select subsets play an important role in the manufacturing process of CAR T cells, particularly when the goal is to increase the representation of a predefined cell population within the final product. Enrichment of specific CD8^+^ and CD4^+^ subsets has been explored as a strategy to enhance the desired characteristics of CAR T cells (Fraietta et al., 2018; Lee et al., 2018; Shah et al., 2020a; Kim-Hoehamer et al., 2023). As each cellular subset within the CD8/CD4 lineage present unique functional characteristics, the identification of populations responsible for improved responses has been pursued as a mechanism of therapeutic improvement. For example, removal of CD4^+^ T cells with enhanced regulatory capabilities, such as regulatory T cells (T_reg_), results in increased overall antitumor activity (Onda et al., 2019). By incorporating these isolation and depletion techniques into the manufacturing process, researchers aim to optimize the composition and functionality of CAR T cells. An approach that has been shown to dictate efficacy of CAR T cells is the infusion of products with pre-defined ratios of select T cells. Specifically, less differentiated subsets (T_N_ and T_CM_) of CD4^+^ and CD8^+^ CAR T cells were more effective in target eradication than those from effector memory (T_EM_) subpopulations, contributing to the correlation between antitumor activity and peak proliferation of CD4^+^ and CD8^+^ CAR T cells (Sommermeyer et al., 2016). As each transduced T cell subset offers unique and sometimes synergistic antitumor functions, the administration of CAR T cell products that consider the proportion of each subset display greater antitumor activity and improved progression-free survival (Turtle et al., 2016; Gardner et al., 2017).

Prior to enrichment of specific CD8^+^ and CD4^+^ T cell subsets, an initial characterization of these cells in the aphaeresis product is important to determine whether enrichment would have any added benefit. If the pre-manufactured product contains high frequencies of terminally exhausted T cells, other approaches, such as allogeneic PBMCs from a healthy donor (Graham et al., 2018; Young et al., 2022), may need to be considered. If an allogenic approach is necessary, additional considerations must be taken to minimize the adverse effects of highly reactive CAR T cells and potential allogenic responses. One approach that has shown promise in these scenarios is the depletion of CD45RA-expressing naïve-like cells from donor PBMCs. This depletion strategy removes T cells possessing a broader T cell receptor (TCR) repertoire that can cross-react with antigens expressed by the recipient (Bleakley et al., 2015), but it also preserves the long-lived functional capabilities of differentiated memory-like T cells that have more restricted TCR repertoires that minimize allogeneic responses (Wherry et al., 2003; Klebanoff et al., 2005). Recent advancements in developing good manufacturing practices (GMP) for CD45RA + cell depletion have been described, facilitating its potential application in CAR T cell manufacturing (Zheng et al., 2018). This has been further tested in the treatment of subjects with lymphocytic leukemia, where the graft-versus-leukemia (GVL) effect is optimized while reducing the risk of graft-versus-host disease (GVHD) (Dutt et al., 2007; Biernacki et al., 2020; Kim-Hoehamer et al., 2023; Naik and Triplett, 2023).

### CAR T cell in-vitro activation

Effective expansion of T cells requires adequate, sustained, and sequential activation signals through a series of surface receptors. The primary signal for T cell activation results from the engagement of the TCR with its ligand (signal 1) and the magnitude of T cell activation and effector function is primarily driven by engagement of costimulatory receptors (signal 2), such as CD28, 4-1BB, ICOS (Bretscher, 1999; Chen and Flies, 2013). This activation triggers the internal proliferative program of T cells, leading to their clonal expansion (Noak et al., 2021).

As specific doses of CAR-expressing T cells within the manufactured product have been shown to have greater clinical efficacy, this activation step can help achieve sufficient numbers of CAR T cells. Various methods of expansion including the use of cell-, antibody-, bead-, and polymer-based activation have been tested with differing levels of success (Gee, 2018; Abou-el-Enein et al., 2021). Activation strategies using cell-based approaches tend to mirror normal immune synapse interactions and drive efficient expansion, but this approach requires greater technical skill, is very laborious, and can be problematic due to having to source sufficient numbers of cells (Sasawatari et al., 2006; Schmidts et al., 2020). An alternative strategy, and one of the most common in current clinical manufacturing workflows, involves the use of super-paramagnetic anti-CD3/CD28 antibody-coated beads, such as Dynabeads™, which provide a reproducible source of T cell activation (Barrett et al., 2014; Gargett and Brown, 2015; Vormittag et al., 2018). One drawback of using Dynabeads™ for activation is the need to remove the beads once sufficient numbers of CAR T cells are obtained, which extends the vein-to-vein infusion time. Newer alternatives, such as a polymeric bio-degradable nanomatrix impregnated with anti-CD3/CD28 monoclonal antibodies, eliminate the added step of bead removal, expediting the manufacturing process of CAR T cells and resulting in a less differentiated composition of cells (Gargett et al., 2019). It should be noted that the use of such nanomatrices do however increase the overall cost of manufacturing (Gee, 2018). While T cell activation is often a necessary step for introducing constructs using retroviruses, newer protocols aim to eliminate cell activation altogether to reduce the manufacturing time and generate cells with less exhausted phenotypes (Yang et al., 2020; Ghassemi et al., 2022). A recent manufacturing protocol that performs same day T cell isolation using CD3/CD28 beads and next-day lentiviral transductions aims to reduce vein-to-vein time and address the *ex-vivo* culturing time required during CAR-T manufacturing. A benefit of this protocol is that it leads to greater T cell stemness in the CAR T cell product, which increases cellular persistence following infusion (Ghassemi et al., 2018). Clinically, CAR-T cells manufactured using the next-day manufacturing process displayed superior expansion and a greater proportion of patients achieved favorable responses compared to conventional CAR T cell manufacturing (Jiang et al., 2020; Zhang C. et al., 2022; Yang et al., 2022).

### CAR construct selection considerations

Development of constructs with unique target specificities and distinct intracellular signaling domains also impacts the extent of T cell activation, expansion, and differentiation (Ghorashian et al., 2019; Mezősi-Csaplár et al., 2023). While first-generation CAR T cells consisted of an extracellular binding domain, transmembrane, and intracellular signaling domain, the lack of expansion in these early CAR T cells has lead researchers to invest significant effort towards incorporating and modifying specific domains in newer iterations with the hope of driving greater expansion and function (Sadelain et al., 2009). Second and third generation CARs incorporate either single or dual costimulatory signaling domains respectively and intend to recapitulate native co-stimulation in T cells, which leads to enhanced intracellular signaling and greater T cell expansion and potency (van der Stegen et al., 2015; Feins et al., 2019; Subklewe et al., 2019; Shah et al., 2020b). The latest (fourth) generation of CARs constructs takes the previous principles of having co-stimulatory domains and incorporates further genetic modifications such as additional co-stimulatory ligands or transgenes for cytokine secretion (Subklewe et al., 2019; Abken, 2021). These latest generation of CAR T cells seek to expand the utility of CARs beyond just target antigen recognition and provide additional avenues to engineer CAR T cells with additional capacities, such as altering the local tumor microenvironment. Clinically, each new iteration of CAR construct is met with challenges, with earlier generations presenting with increased relapsed rates and later generations resulting in greater toxicities (Park et al., 2016; Cappell and Kochenderfer, 2023).

### Methods for introducing CAR constructs to T cells

Traditionally, viral-based techniques have been used for CAR T cell production, which involves delivering CAR genes into T cells using retroviral or lentiviral vectors (Rosenberg et al., 1990; Scholler et al., 2012; Gardner et al., 2017; Marcucci et al., 2018). These viruses randomly integrate their genetic material into the host cell genome, enabling long-term expression of the CAR. This approach has demonstrated remarkable success in generating CAR T cells with potent antitumor activity and has led to significant advancements in clinical settings. However, viral-based methods come with challenges. Safety concerns arise due to the potential for viral integration causing insertional mutagenesis or activating oncogenes (Scholler et al., 2012; Cavazza et al., 2013). Manufacturing viral vectors is complex, time-consuming, and expensive, hindering scalability and clinical accessibility. Moreover, regulatory requirements associated with viral vectors add further obstacles to the development and commercialization of CAR T cell therapies.

To address these limitations, non-viral synthetic biology methods have emerged as alternative approaches for CAR T cell production. RNA-based delivery systems have gained attention for achieving transient CAR expression without genomic integration (Rabinovich et al., 2009; Zhao et al., 2010; Soundara Rajan et al., 2020). Messenger RNA (mRNA) encoding the CAR construct is introduced into T cells, leading to efficient CAR expression. This method allows for fine-tuned control of CAR expression levels by adjusting RNA dosage and duration of expression, thereby reducing off-target effects. Unlike DNA integration, RNA offers reversible and transient control over gene expression, enabling CAR expression to subside over time and without constitutive expression in progeny cells after CAR T cell expansion. This adaptability may facilitate adjustments of CAR expression in response to patient conditions, optimizing therapy efficacy and minimizing risks for more tailored outcomes. Furthermore, it is important to highlight the potential impact of the controlled expression on mitigating cytokine release syndrome (CRS), immune effector cell-associated neurotoxicity syndrome (ICANS) (Gauthier and Turtle, 2018; Siegler and Kenderian, 2020), and tonic signaling leading to the exhaustion of T cells (Lamarche et al., 2023)–all of these detrimental effects have been associated with CAR overexpression resulting from viral-based technologies. This reduction in potential adverse effects could contribute significantly to the safety and efficacy of CAR T cell therapies. However, repeated dosing is necessary for sustained CAR expression, posing challenges for large-scale manufacturing and clinical implementation (Moretti et al., 2022).

DNA transposon systems offer a non-viral method for stable CAR integration into the T cell genome. DNA transposons are DNA segments flanked by inverted repeats that can “jump" into the host genome with the assistance of a transposase enzyme. Incorporating the CAR gene within a transposon allows for its stable integration, providing long-term CAR expression without relying on viral vectors. The transposon, containing the CAR gene, is flanked by inverted terminal repeats (ITRs) and delivered as a (nano-) plasmid or minicircle DNA. The transposase enzyme assists in integrating the transposon into the acceptor DNA at specific target sites, such as -TA- (Sleeping Beauty) (Ivics et al., 1997) or -TTAA- (PiggyBac) (Gogol-Döring et al., 2016) sequences. While the integration may not be precisely targeted, it can occur in preferred insertion sites known as “safe harbors" (Querques et al., 2019) and offers long-term CAR expression. Researchers are actively exploring different transposon systems such as the Sleeping Beauty and piggyBac systems (Kebriaei et al., 2016; Magn et al., 2020; Zhang X. et al., 2022) to enhance their performance and suitability for clinical applications. Although DNA transposon systems offer improved safety, optimizing their efficiency and minimizing off-target effects are ongoing challenges. The CARTELL trial, which employed PiggyBac technology for the treatment of relapsed/refractory B cell malignancies, encountered a concerning outcome as two out of ten patients developed lymphoma (Micklethwaite et al., 2021). This outcome has raised safety concerns surrounding the use of PiggyBac and transposon/transposase-based cell therapies for the treatment of B cell malignancies. The development of lymphoma may be related to the manufacturing process or an increase in global copy number changes observed in the products. In response to these safety concerns, there is a recognized need for the development of enhanced preclinical genotoxicity models and optimization strategies. Transcription activator-like effector nucleases (TALEN) and CRISPR/Cas9-mediated genome engineering provide avenues for site specific modification in T cells. TALENs, composed of hybrid molecules merging DNA recognition proteins (transcription factors) with an endonuclease, utilize TAL units of 33–35 amino acids to recognize a single base pair on genomic DNA. Linking several TALs with an endonuclease creates a site-specific TALEN, a crucial tool for precision engineering in the development of CAR-T cells (Jo et al., 2022). CRISPR/Cas9-mediated genome engineering, on the other hand, uses the CRISPR/Cas9 (Jinek et al., 2012) system to introduce precise modifications in the T cell genome (Dimitri et al., 2022). By designing a guide RNA (gRNA) specific to the target site, the Cas9 enzyme can cleave the DNA, triggering DNA repair mechanisms and allowing for gene modifications such as CAR integration or endogenous gene knockouts (Eyquem et al., 2017; Roth et al., 2018; Kamali et al., 2021; Shy et al., 2021). This method offers versatility and precision in modifying the T cell genome, enabling customized CAR designs and improved therapeutic efficacy. Nevertheless, the application of CRISPR technology is not without risks, and ongoing research is dedicated to addressing concerns such as reducing off-target effects, managing mosaicism, addressing potential chromosome translocations, and refining DNA repair processes. Current research efforts are focused on enhancing efficiency, mitigating risks, and ensuring the safety of CRISPR-edited cells, with the goal of providing effective gene engineering strategies to alter the T cell’s genome for therapeutic purposes.

Recently, an RNA-guided endonuclease, Fanzor (Fz) (Saito et al., 2023) protein, has been identified from the eukaryotic system and can be used for genome editing. Although this newly identified protein has not yet been used for the generation of CAR T cells, its eukaryotic origin and relatively small size compared to Cas9/12 make it an attractive starting point for further development and use for cellular engineering. Engineering strategies such as systematic mutagenesis and guide RNA engineering combined with in-depth screening of more Fanzors could further improve their genome editing performance, highlighting the potential of Fzs for cellular engineering.

The traditional viral-based methods, while successful, face challenges related to safety, scalability, and manufacturing complexity. Non-viral methods like RNA-based delivery systems and DNA transposon systems offer advantages in terms of controlled expression and stable integration, respectively. TALENs and CRISPR/Cas9 provide greater precision in genome editing, but CRISPR/Cas9 can also present specific risks such as chromosomal translocations. Fanzor protein, although promising, requires further development as this is a relatively new cell engineering strategy.

## Experimental approaches to test functionality for potency testing

As CAR T cell therapy manufacturing generates final products consisting of heterogeneous phenotypes, implementing standardized potency testing, and establishing thorough release criteria before infusion are imperative to ensure safety, efficacy, and consistency of care. Historically, quality control testing has primarily focused on the absence of contaminants like *mycoplasma* and endotoxins (Gee, 2018). However, the evolving field of biologics manufacturing has prompted the FDA and EMA to recognize the need for updated criteria encompassing assays that evaluate whether CAR T cells can mediate antitumor functions through cytotoxic- or cytokine-based mechanisms (Food and Drug Administration, 2019; European Medicines Agency, 2022; European Medicines Agency, 2018b; Gee, 2015; Si et al., 2022). By adopting standardized assays, the safety and efficacy of CAR T therapies can improve, ensuring administration of only high-quality products to patients (Aleksandrova et al., 2019; Roddie et al., 2019). Lastly, the emergence of new technologies enables more efficient pre-infusion testing, facilitating the evaluation of the therapeutic potential of CAR T cells before infusion.

### Secretory profiling

Proteomic characterization of secreted analytes has become a necessary criteria prior to infusion due to the potential risks following CAR T cell infusion is developing CRS and neurotoxicity (Maude et al., 2014; Brudno and Kochenderfer, 2016; Gardner et al., 2019; Hopfinger et al., 2019; Sterner and Sterner, 2021). To mitigate this risk, current release criteria rely on using multi-analyte profiling (xMAP™) which are bulk assays that measure soluble factors from cell cultures through the use of labeled microspheres that capture multiple analytes at a time (Maude et al., 2014; Graham et al., 2019; Melenhorst et al., 2022). Unfortunately, by characterizing cytokine secretion in bulk, these assays provide a global view of CAR T functionality and may not accurately depict the heterogeneous nature of the CAR T cell products and can pose challenges when trying to predict clinical outcome. Newer technologies incorporating single-cell approaches can capture the heterogenous nature of CAR T cells and provide a more granular view of how each cell behaves rather than taking an average measure across all cells. The development of instruments such as Bruker Cellular Analysis’ IsoSpark allows interrogation of the secretory profile of CAR T cells at single cell resolution and has provided useful correlations to be made between the product’s secretome and clinical response (Lu et al., 2013; Mackay et al., 2017; Rossi et al., 2018; Xu et al., 2020; Zurko et al., 2022). Using the Polyfunctional Strength Index (PSI) score, which describes the percentage of polyfunctional single cells in a sample secreting two or more analytes (IsoPlexis, 2023), standardized metrics could be incorporated as part of the release criteria for CAR T cells and may ultimately provide a better measure of clinical response and help minimize the toxicities observed in many patients.

### Cytotoxic profiling

One of the most relevant functions that must be evaluated in the final product is the cells' ability to have sufficient cytotoxicity against antigen-expressing target cells. By using instruments that measure changes in target cell density with either electrical impedance or image-based approaches, one can evaluate the cytotoxic potential in bulk co-culture studies. Impedance-based assays are real-time cell analysis systems that monitor interactions without requiring labels or invasive techniques (Logun et al., 2023). Systems such as Agilent’s xCelligence™ and Axion’s Maestro ™ can indirectly measure processes such as proliferation and cytotoxicity by using specialized microplates with integrated electrodes and measuring electrical impedance (Kiesgen et al., 2021; Lamarche, 2021; Axion Biosystems, 2024). Image-based approaches measure cytotoxicity in real-time, using automated high-resolution imaging and fluorescence analysis (Strietz and Chen, 2022). This technology implemented in instruments such as Sartorius’ Incucyte™, Enrich Biosystem’s Trovo™, and Nanolive’s 3D Cell Explorer™ can track and analyze additional cellular processes over time, including proliferation, cytokine secretion, and cytotoxicity (Zah et al., 2020; Nanolive, 2023; Sartorious, 2019). Both detection approaches provide useful readouts for functional testing and may provide clinically relevant data that could also be incorporated as part of the CAR T cell release criteria prior to infusion. The real-time monitoring capabilities and analytical power of these instruments highlight their value in understanding the dynamic cellular processes occurring between effector and target cells and can help generate data that more accurately reflects how CAR T cells behave *in vivo*.

### Polyfunctional characterization

Most commercially available CAR T cell products implement a one-size-fits-all manufacturing process and often ignore inter-patient differences that may dictate treatment outcome. By incorporating single-cell functional assays using technology capable of measuring multiple functional characteristics in parallel, researchers can comprehensively assess how the isolation and expansion of specific CAR T cell subsets will drive clinical efficacy (Mocciaro et al., 2018; Bronevetsky, 2020; Le et al., 2020; Romain et al., 2022). The recent explosion of various single cell platforms has revolutionized the field of functional biology and has allowed researchers to not only interrogate how cells function over time using single cells or groups of cells, but has also allowed for the isolation of specific subpopulations of cells with unique functional characteristics from a patient’s blood or tissue on a per-patient basis (Bandey et al., 2021; Miwa et al., 2022; Urbani et al., 2022). Commercial technologies that have implemented this type of multiparameter testing, such as CellChorus’ TIMING™ and Celldom’s^®^ CloneXplorer™ platforms, have utilized well-based technology to seed effector and target cells and record effector function using brightfield and fluorescent imaging (Liadi et al., 2015; Celldom, 2022). Other platforms such as Bruker Cellular Analysis’ Beacon™ and Lightning™ systems utilize non-destructive light to sort cells into NanoPen^®^ chambers distributed on OptoSelect™ Chips and have the capability of longitudinally performing both cytokine secretion and cytotoxic assays at the single cell level allowing researchers to identify cells that display the best response to cancer (Bronevetsky, 2020; Berkeley Lights, 2024a). Functional cells of interest can then be isolated for additional expansion or to perform downstream assays, such as transcriptional profiling to identify immune signatures depicting unique functional states (Liadi et al., 2015; Berkeley Lights, 2024b). One drawback is that due to the temporal nature of these assays, the current implementation of these characterizations as release criteria would lengthen the manufacturing time and delay infusion.

### Challenges and next steps

Current advancements in technology and the implementation of in-depth characterization are opening avenues for improving the quality of infused CAR T cell products. The emergence of single-cell functional experiments provide a valuable opportunity to investigate and quantify phenotypic, functional, and transcriptional profiles of single cells. The knowledge gained will result in improved product design and manufacturing processes. Nevertheless, to fully unlock the potential of adoptive immunotherapies in treating refractory diseases, more robust and in-depth cellular characterizations uploaded to public databases will accelerate the improvement of future therapies. Despite the technological advances that help link functional variables and therapeutic efficacy, gaps remain in understanding the impact of previous treatment modalities on CAR T cell efficacy and how other factors, such as the temporal evolution in the tumor cells’ ability to evade T-cell-mediated killing, result in suboptimal responses. To gain insight into these limitations, temporal transcriptional profiling of patient samples at single-cell resolution and longitudinal characterization of CAR T cells pre- and post-infusion has helped validate unique gene-expression profiles that gave rise to highly functional post infusion phenotypes (Wilson et al., 2022). Despite increased efforts to characterize and validate CAR T cell products, there are inherent risks that come with any cell-based engineering strategy. Recently, the FDA has begun evaluating whether greater regulatory action is needed for CAR T cell therapies as potential risks of developing T cell malignancies and secondary hematological cancers have been reported in patients that have received this line of therapy. While the current consensus appears to be that the benefits of CAR T cell therapy outweigh the potential risks for patients with cancer, these recent cases highlight the need for improved strategies aimed at preventing random integration of CAR constructs. More stringent characterization of CAR T cell products may also help identify cases where CAR-associated malignancies arise (FDA, 2023a; Harrison et al., 2023; Mullard, 2023).

By optimizing specific steps in the manufacturing of CAR T cells and incorporating some of the potency assays highlighted in this review, patients will begin receiving a more robust product with a greater proportion of cytotoxic CAR T cells that have longer persistence. The next steps would be to consider whether combination therapies using other therapeutic modalities or helping to overcome an immunosuppressive tumor microenvironment would increase success rates (Huang et al., 2022). An added benefit in building more comprehensive multimodal datasets is that this information can also help inform whether specific manufacturing steps should be altered and what the likely clinical outcomes would be. Finally, these data could also allow for a greater ability to identify individuals likely to respond to CAR T cell therapy using specific biomarkers or help physicians decide whether a different course of treatment is warranted.

## In silico approaches to investigate CAR T-cell function and predict therapeutic responses

As outlined in previous sections, there are several possible variables introduced during CAR T cell manufacturing which will influence the CAR T cell product. It can be extremely difficult to interrogate how these decisions ultimately impact product functionality and patient responses using currently available experimental techniques alone. Recently, there has been an explosion of computational models aiming to tease apart relationships between the CAR T cell product manufacturing process, product functionality, and the resulting therapy responses to predict how manufacturing variables should be modulated to improve overall clinical response. We direct the reader to substantial prior works for more in-depth reviews on computational models and modeling methods in CAR T cell therapy (Nukala et al., 2021; Qi et al., 2022). In this section, we will review notable approaches and discuss their potential to inform CAR T cell manufacturing and improve the clinical efficacy of the therapy.

### Computational models for characterizing CAR T cell function

Changes in CAR insertion methods can modulate CAR surface expression and avidity toward target antigens leading to changes in CAR T cell functionality. Mechanistic models based on kinetic-proofreading concepts have been developed to investigate intricate relationships between antigen densities, CAR surface expression and ligand-receptor binding rates. These models demonstrate that it is possible to control CAR T cell product functionality through tuning CAR receptor expression and antigen binding rates in relation to tumor cell antigen densities (Ha et al., 2018). Additionally, these models hypothesize that it may be possible to restrict CAR T cell activity toward tumor cells and mitigate off-target effects (cytolysis of healthy cells) by tuning avidities (Ha et al., 2018; Rajakaruna et al., 2023). These models offer an intriguing design tool for optimizing the performance of CAR T cell products in specific tumors, and potentially in specific patients, as a function of tumor antigen densities.

Additionally, as each domain in the CAR construct can be switched and modified (Mazinani and Rahbarizadeh, 2022), exactly how these changes modulate intracellular signaling and product functionality are important to consider. A mechanism-based (mechanistic) ordinary differential equation (ODE) model has been developed to study the intricate kinetics of CAR receptor and intracellular extracellular signal-regulated kinase (ERK) activation in the presence and absence of CD28 co-stimulation supported by protein phosphorylation kinetics derived using phosphoproteomics (Rohrs et al., 2018; Rohrs et al., 2020). Insights from the model can be used to understand the mechanisms of CAR signal propagation in CAR T cells and help develop hypotheses for optimizing CAR designs to improve product functionality through modulating the CD3ζ and CD28 signaling domains. It is important to note, however, that models such as these are difficult to develop and are limited to study specific construct designs and signaling contexts which ultimately limit their utility. As the field is quickly advancing toward widely variable CAR constructs (Mazinani and Rahbarizadeh, 2022), more robust computational methods need to be developed to enable optimization techniques.

### Computational models for CAR T cell therapeutic responses

Frequently described as a “living drug,” therapy dynamics for CAR T products are exceptionally challenging to model using established pharmacokinetic (PK) and pharmacodynamic (PD) approaches used for small molecule drugs. One key complication is that CAR T cells continue to differentiate during manufacturing and after infusion leading to variable kinetics of cytotoxicity and proliferation. Further complicating model development are the inter-product and inter-patient variabilities in CAR design, differences in manufacturing conditions and patient-specific baseline T cell phenotype and function following pretreatment, tumor burden and antigen expression, and tumor microenvironments, which collectively impact therapeutic response. Nonetheless, considerable efforts have been directed at developing computational models for therapy response. Several proposed models use ODE-based approaches with simple predator-prey (Lotka-Volterra) mechanisms for modeling dynamics between CAR T cells and tumor cells (Hardiansyah and Ng, 2019; Sahoo et al., 2020). Additionally, mathematical relationships derived from enzyme kinetics and empirical relationships have also been used to describe these interactions (Stein et al., 2019; Singh et al., 2020; Liu et al., 2021). Once formulated, therapy response models can be used to explore how various facets of the therapy ultimately influence response. Additional variables such as a patient’s age, disease burden, type and duration of bridging therapy, lymphodepletion regimen, and whether prior lines of therapy have been administered can be incorporated into these models. Additionally, models can give insight into the impact of dosing and lymphodepletion regimens on resulting therapy kinetics (Hardiansyah and Ng, 2019; Stein et al., 2019; Kimmel et al., 2021; Owens and Bozic, 2021). Parameters derived from these models can help elucidate kinetic rate constants for target cytolysis and CAR T cell proliferation (Kimmel et al., 2020; Liu et al., 2021; Amini et al., 2022; Lickefett et al., 2023). These parameters can potentially be used in conjunction with functional assays and other variables to help evaluate future products.

Importantly, models have also begun to describe how different phenotypic and functional states of the CAR T cell product can shape *in vivo* expansion and tumor clearance (Stein et al., 2019; Mueller-Schoell et al., 2021; Paixão et al., 2022; Kirouac et al., 2023). With further validation against clinical and experimental datasets, these models have the potential to be used to define additional biomarkers for predicting the survival of patients receiving CAR T cell therapy based on assessing product composition pre-infusion and tracking changes in CAR T cell compositions post-infusion (Mueller-Schoell et al., 2021; Paixão et al., 2022). It may also be possible to use this information to develop optimal CAR T cell product compositions to improve clinical outcome which can then be used to further optimize variables at different steps of manufacturing. Additional applications for therapy response models are to predict the occurrence of serious side-effects associated with therapy administration such as CRS and neurotoxicity. Physiologically based PK and PD (PBPK/PD) models are especially useful in understanding the possible CAR T cell distribution after they are infused into the body and help develop exposure-response relationships for neurotoxicity potentially by proxy of CAR T trafficking into non-tumor compartments (Singh et al., 2020). This information may provide additional insight into which subsets of CAR T cells to deplete to prevent most of the cells trafficking to non-tumor compartments. CRS can also be examined through determining the rate of production of key cytokines post therapy administration (either by CAR T cells or other sources in the body). Models can help predict the dynamics of cytokine release to direct timing of intervention measures and offer methods to demonstrate the interaction between prophylactic drugs and CAR T cells (Hardiansyah and Ng, 2019; Stein et al., 2019; Zhang Z. et al., 2022). These models in conjunction with secretory profiling methods discussed in previous sections may provide additional depletion strategies to avoid CRS.

One major limitation of the ODE-based models for CAR T cell therapy overviewed thus far is that they require preexisting knowledge of physiological mechanism and robust datasets to estimate parameters which inherently limits the number of variables the models can include. To sidestep some of these obstacles, alternative approaches utilizing logic-based and agent-based techniques are also being explored. These models are able to capture more of the complexities observed in CAR T cell therapy such as relationships between key cytokines, CAR T cell subsets (CD4, CD8), intracellular signaling, and environmental factors to develop more comprehensive models (Selvaggio et al., 2022; Prybutok et al., 2022; Shah et al., 2023). These approaches are attractive for exploratory studies for CAR T cell therapy to help quickly interrogate the influence of multiple variables and drive hypotheses for fine tuning manufacturing conditions. It remains to be seen whether these models can be validated and/or provide utility beyond ideation.

It should be noted that therapy response models developed to-date and reviewed here either aim to study the system on a theoretical level or are otherwise descriptive of specific patient or patient-cohort therapy responses considered for developing the model. Given the wide range of factors during CAR T cell manufacturing that control possible therapy responses, models currently offer limited predictive and prognostic value for future products and patients as critical parameters used for the models cannot be derived prior to therapy administration and will generally differ on a product and patient basis. Using *in vitro* assays to drive modeling can offer a potential avenue to both validate physiological mechanisms used in models and to derive parameters necessary for model simulations ahead of therapy administration (Sahoo et al., 2020; Kirouac et al., 2023; Li et al., 2023b). Experimental techniques reviewed in previous sections may offer modelers additional methods with which to support model development and help enable clinical applications for these models.

### Challenges and next steps

**FIGURE 2:**
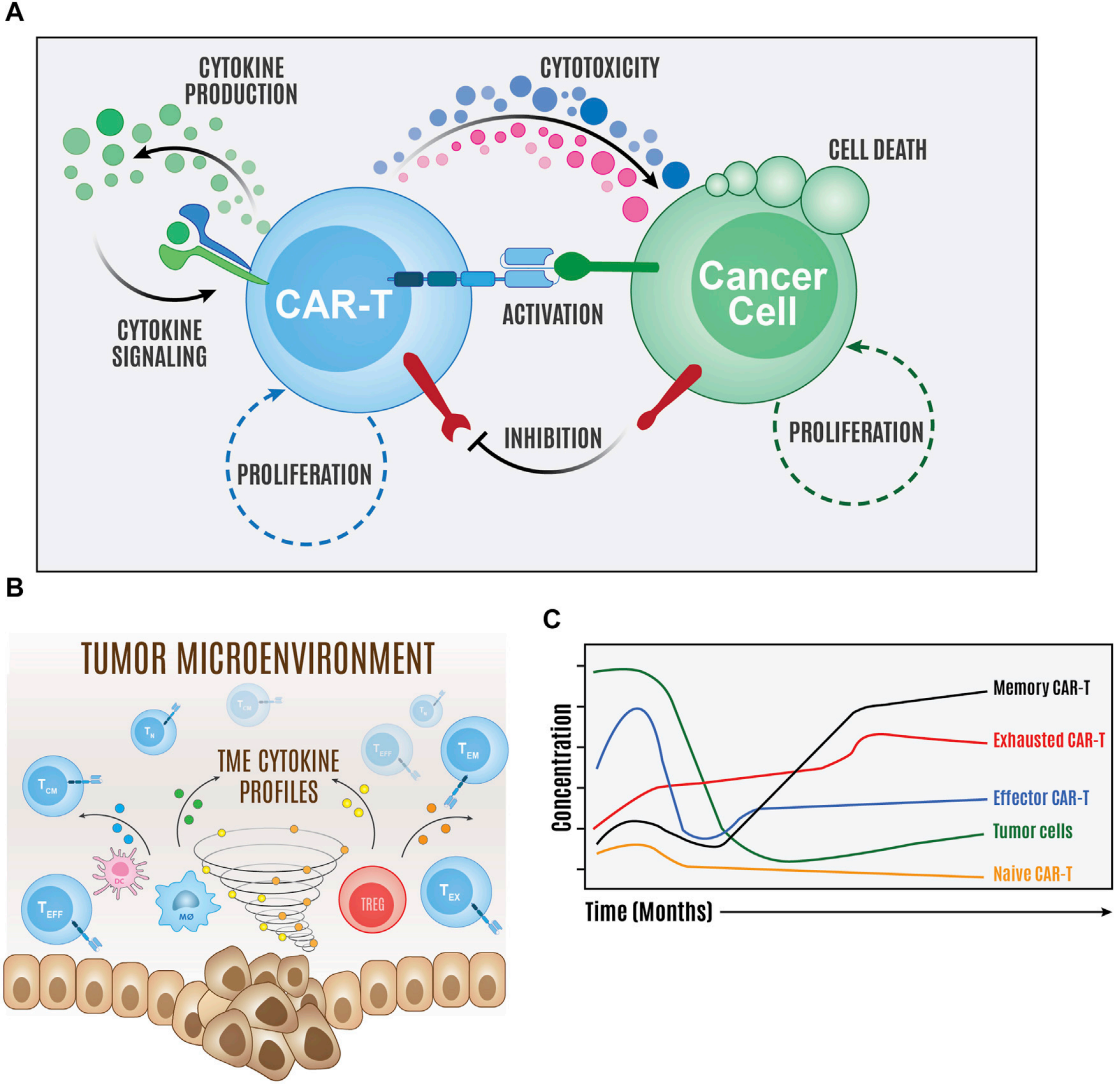
Overview of computer simulations of CAR T cell therapy. Computational models of the major components of the molecular interaction network **(A)** and population cellular states and environment **(B)** can be created to simulate and predict therapy temporal dynamics **(C)**.

One of the major challenges in developing clinically relevant computational models for CAR T cell therapy is the availability of well characterized and robust datasets that can be used to formulate these models. Current experimental and clinical datasets fall short in routinely and comprehensively characterizing CAR T cell products, subset compositions, cytolysis and proliferation rates, biodistribution, and receptor expression. There are similar limitations for tumor cells due to the lack of data on proliferation rates and antigen expression. This poses challenges when trying to develop models to determine how key manufacturing variables will influence response. With larger and better curated datasets, the methods used to develop the models reviewed here for describing CAR T cell product functionality and therapy response have the potential to inform new manufacturing protocols and enable predictive and precision medicine in CAR T cell therapy. There is opportunity to utilize these preliminary works to provide rationale for the collection of appropriate datasets, especially where the impact on clinical outcome is not immediately clear. The goal is to advance and begin implementing computational techniques that may offer avenues to ultimately improve therapeutic effectiveness in the long-term. An overview of how computational models can be used to simulate CAR T cell therapy response and temporal dynamics is depicted in Figure 2. With the recent expansion and incorporation of artificial intelligence (AI) in systems biology and medicine, there is growing interest in determining how to best apply AI to improve cell-based manufacturing. Development of automated AI-driven CAR T cell manufacturing processes have been proposed and aim to decrease the cost, reduce the amount of manual labor, and improve the efficacy of CAR T cell products (Hort et al., 2022). These computational approaches provide additional opportunities to rapidly advance the next line of CAR T cell products.
